# Sexual dimorphisms of the olfactory system are conserved across bumblebee species

**DOI:** 10.1016/j.isci.2026.118037

**Published:** 2026-10-28

**Authors:** Jorn Oppersma, Wenke E, W. Carolina Gomez-Ramirez, Iman Muktar, Timothy Wise, Miranda Bolton, Shuang Chen, Amir Atapour-Abarghouei, Carlo Polidori, Olena Riabinina

**Affiliations:** 1 Department of Biosciences, Durham University, Durham, UK; 2 Department of Computer Science, Durham University, Durham, UK; 3 Department of Environmental Science and Policy (ESP), University of Milan, Milan, Italy; 4 Evolution, Genomes, Behaviour and Ecology, Université Paris-Saclay, Gif-sur-Yvette, France; 5 Department of Cell and Developmental Biology, University College London, London, UK; 6 Institute for the Physics of Living Systems, University College London, London, UK

**Keywords:** antennal lobe, sensilla, automatic segmentation, comparative anatomy

## Abstract

Bumblebees are integral to ecosystems as crucial pollinators. However, neuroscience has kept its focus on studying workers of commercially available species: *Bombus terrestris* (Europe), *B*. *impatiens* (North America), and *B*. *ignitus* (Asia). Here, we conducted a detailed comparative study of the brain and antennal anatomy in wild worker and male bumblebees of 6 common European species (*B. terrestris*, *B. pascuorum*, *B. pratorum*, *B. lapidarius*, *B. hypnorum*, and *B. lucorum*), with specific focus on their peripheral and central olfactory system. We found striking sexual dimorphisms that remain consistent across species and may underlie the differences in foraging preferences in bumblebees. We also developed a bespoke machine learning-based pipeline for automatic segmentation of confocal images of antennal lobes, which greatly speeds up the image analysis.

## Highlights

• Workers have larger antennal lobes, more glomeruli and olfactory sensilla than males
• There are species-specific differences in the antennal lobe organization
• The sex dimorphisms in the antennal lobe anatomy are consistent across species
• Olfactory system dimorphisms correlate with the foraging repertoire

## Graphical abstract

**Figure:**
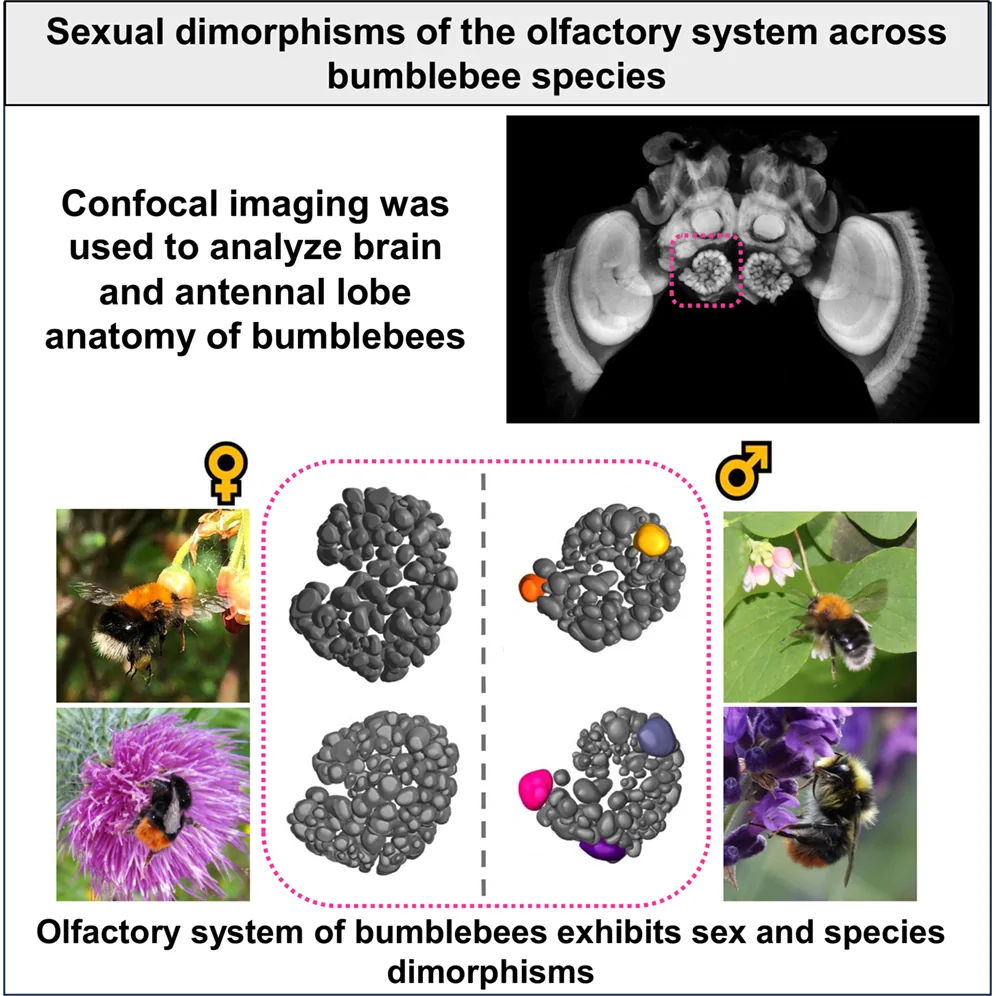

## Teaser

Zoology; Entomology; Neuroscience; Machine learning

## Introduction

Bumblebees are efficient and agriculturally important pollinators that are currently threatened by climate change and other human activities, causing rapid population declines.^1^^,^^2^^,^^3^ However, neuroanatomy and neurophysiology of bumblebees have been focused on the species that are commercially available in Europe, North America, and Asia, and can be bred in laboratory settings—*Bombus terrestris*, *B. impatiens*, and *B. ignitus*.^4^^,^^5^^,^^6^^,^^7^^,^^8^ Little is known about the brain anatomy and function of other bumblebee species, and how they link to their foraging preferences.^9^^,^^10^^,^^11^ Moreover, the studies on the brain anatomy of male bumblebees have been very limited,^12^^,^^13^ and their central olfactory system has not been described previously.

Here, we focused on wild worker and male bumblebees of 6 species that are generalists, sympatric in the North-East England and occur commonly throughout Europe: *B. terrestris*, *B. pascuorum*, *B. pratorum*, *B. lapidarius*, *B. hypnorum*, and *B. lucorum*. These species represent both phylogenetically close (*B. terrestris* and *B. lucorum*) and distant (e.g., *B. terrestris* and *B. pascuorum*) species and cover 4 subgenera of *Bombus*.^14^ The studied species are all primitively eusocial with an annual cycle, but show some differences in their behavioral ecology: *B. lucorum* and *B. lapidarius* nest in the ground, often re-using rodents’ old tunnels; *B. pratorum* and *B. hypnorum* nest in above-ground cavities in trees or in birds’ nest-boxes, and *B. pascuorum* and *B. terrestris* are flexible and can nest both below and above ground. Colony size is also variable, ranging from about 100 to 200 workers in *B. pratorum* and *B. pascuorum* to around 400 in *B. terrestris* and *B. lucorum*.^15^ All species are polylectic, foraging on a wide range of plant species, although local specific preferences may emerge.^16^ It is thus feasible that these ecological differences between species are mirrored by species-specific adaptations of their sensory systems.

We asked whether the anatomy of the olfactory system at the level of antenna and the primary olfactory brain center, the antenna lobe (AL), differs across species and sexes (workers and males), and whether it reflects the diversity of plants that the bees forage on.

## Results

### Brain and antennal lobe volumes

To investigate the brain anatomy of bumblebees, we have immunostained their brains with a synaptic marker and imaged the brains using confocal microscopy (Figures 1A and 1B). We found that total brain volumes did not exhibit sex- or species-specific dimorphisms (Figure 1C, mean ± STD: (6.5 ± 1.4) × 10^8^ mm^3^, *n* **=** 42, two-way ANOVA: *F_sex* (1, 35) = 2.4, *p_sex* = 0.13, *F_species* (5, 35) = 1.4, *p_species* = 0.25). In contrast, absolute AL volumes were consistently higher in workers compared with males, and also differed across species, ranging from (0.6–1.5) × 10^7^ mm^3^ (Figure 1D, two-way ANOVA: *F_sex* (1, 35) = 23.3, *p_sex* < 0.0001, *F_species* (5, 35) = 4.8, *p_species* = 0.002). Absolute AL volumes strongly correlated with the overall brain volumes (Figure 1E, *simple linear regression model*, *F* = 20, *p* = 0.0001, R^2^ = 0.4). Relative AL volumes, calculated as a ratio [*absolute AL volume/brain volume*] for each specimen, remained sexually dimorphic but did not differ significantly between species (Figure 1F, two-way ANOVA: *F_sex* (1, 35) = 5.3, *p_sex* < 0.027, *F_species* (5, 35) = 0.9, *p_species* = 0.49), constituting on average 0.017 ± 0.003 (mean ± STD) of the total brain volume. Relative AL volumes had a negative correlation with the total brain volume (Figure 1G, *simple linear regression model*, *F* = 8.6, *p* = 0.006, R^2^ = 0.22), implying that absolute AL volume does not scale isometrically with the overall brain volume.

**Figure 1:**
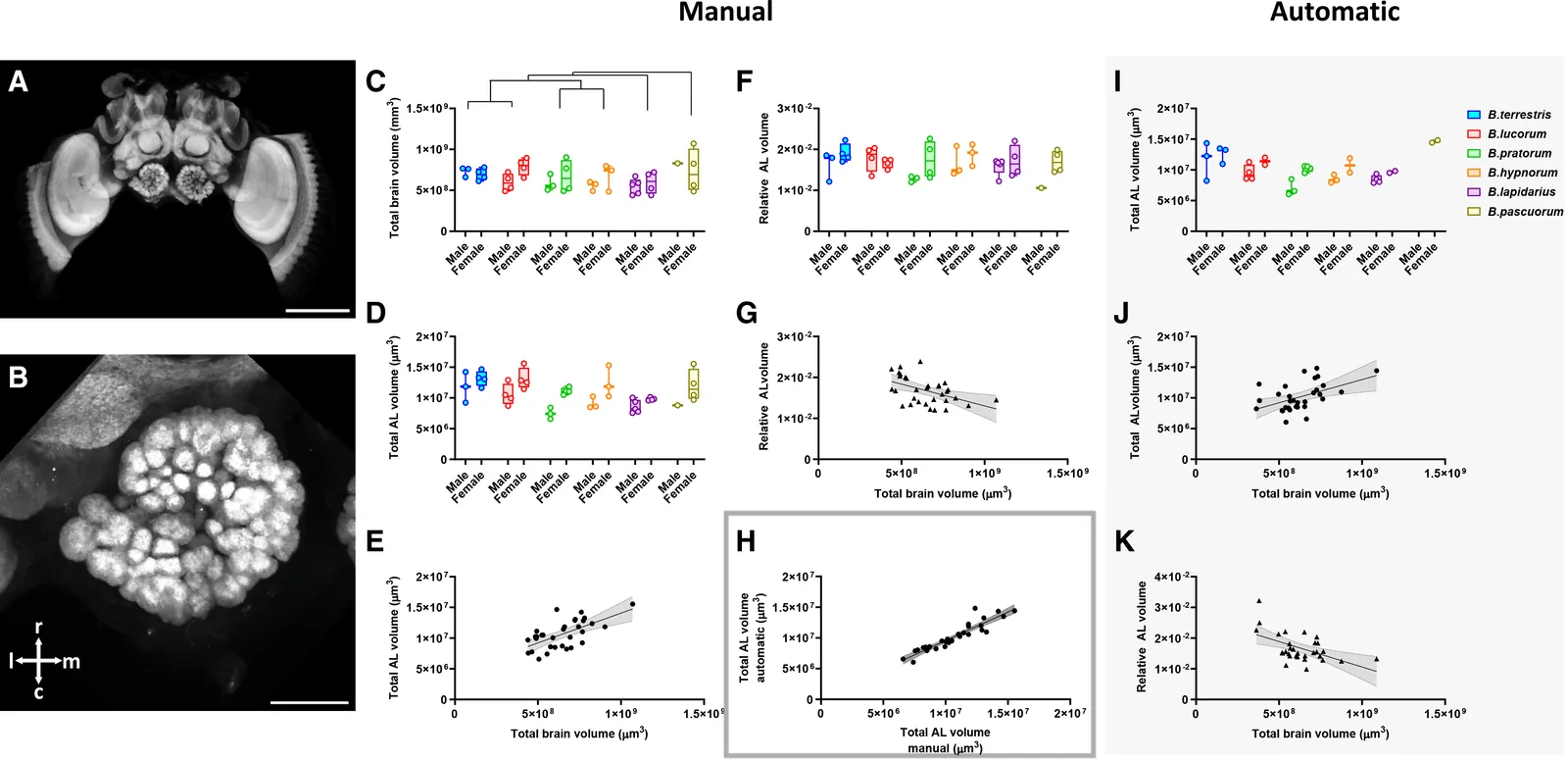
Characterization of brains and ALs (A) Maximum intensity projection of a *Bombus hypnorum* brain confocal image stack. (B) Maximum intensity projection of a *B. hypnorum* antennal lobe (AL) confocal image stack. (C and D) Total brain volume (C) and total antennal lobe (AL) volume (D) of males (M) and females (F) of *B. terrestris* (blue; M: *n* = 3; F: *n* = 4), *B. lucorum* (red; M: *n* = 5; F: *n* = 4), *B. pratorum* (green; M: *n* = 3; F: *n* = 4), *B. hypnorum* (orange; M: *n* = 3; F: *n* = 3), *B. lapidarius* (purple; M: *n* = 5; F: *n* = 4), and *B. pascuorum* (yellow; M: *n* = 1; F: *n* = 3). Species are ordered based on phylogenetic relationships, as shown above (C). (E) Correlation of total AL volume and total brain volume. (F) Relative AL volume across males (M) and females (F) of *B. terrestris* (M: *n* = 3; F: *n* = 4), *B. lucorum* (M: *n* = 5; F: *n* = 4), *B. pratorum* (M: *n* = 3; F: *n* = 4), *B. hypnorum* (M: *n* = 3; F: *n* = 3), *B. lapidarius* (M: *n* = 5; F: *n* = 4), and *B. pascuorum* (M: *n* = 1; F: *n* = 3). (G) Correlation of relative AL volume and total brain volume. (H) Correlation of the total AL volume using automatic segmentation and the total AL volume with manual segmentation. (I) Total antennal lobe volume using automatic segmentation of males (M) and females (F) of *B. terrestris* (M: *n* = 3; F: *n* = 3), *B. lucorum* (M: *n* = 4; F: *n* = 2), *B. pratorum* (M: *n* = 3; F: *n* = 4), *B. hypnorum* (M: *n* = 3; F: *n* = 2), *B. lapidarius* (M: *n* = 4; F: *n* = 2), and *B. pascuorum* (M: *n* = 0; F: *n* = 2). (J) Correlation of the automatically segmented total AL volume and total brain volume. (K) Correlation of the automatically segmented relative AL volume and total brain volume. Scale bars, (A) 500 μm and (B) 100 μm. v, ventral; m, medial; d, dorsal, l, lateral. Box-whisker plots (C, D, F, and I): the box indicates 25^th^, 50^th^, and 75^th^ percentiles, the whiskers extend to min and max values. The lines and the shaded areas (E, G, H, J, and K) represent simple linear regression lines with 95% confidence interval.

To reduce potential bias during manual image analysis, and to test the feasibility of a faster automated approach, we developed a pipeline for automatic volume calculation of the whole brains and ALs (see STAR Methods for details). The pipeline was evaluated in an unattended fixed-parameter configuration, in which the same parameter set was applied to all 42 image stacks without sample-specific adjustment. Thirty-two of the 42 stacks (76%) fell within this operating range and were successfully processed. In the remaining 10 stacks, weak or spatially heterogeneous signal-to-background contrast reduced the separability of the AL foreground and local intensity gradients at some interglomerular boundaries, while elevated high-frequency noise destabilized foreground and marker extraction. Because these operations precede the marker-controlled 3D watershed, errors at this stage propagate to incomplete or unreliable foreground masks and/or spurious markers, which subsequently result in unstable segmentation or over-segmentation. We therefore treated these 10 stacks as failures of unattended fixed-parameter processing. Addressing them automatically would still have been possible but would have required sample-specific parameter adjustment and manual quality control, which was outside the intended fully automated workflow. The automatic AL volume measurements were in close agreement with the volumes obtained by manual segmentation (Figure 1H, *simple linear regression model*, *F* = 188.5, *p* < 0.0001, R^2^ = 0.86), and were also sex- and species-specific (Figure 1I, two-way ANOVA: *F_sex* (1, 25) = 15.6, *p_sex* = 0.0006, *F_species* (5, 25) = 7.9, *p_species* = 0.0001). The correlations between absolute (Figure 1J, *simple linear regression model*, *F* = 8.96, *p* = 0.0056, R^2^ = 0.24) and relative (Figure 1K, *simple linear regression model*, *F* = 11.4, *p* = 0.002, R^2^ = 0.28) AL volumes and total brain volumes were also apparent in the automatic quantification results.

### Manual and automatic segmentation and quantification of the antennal lobe glomeruli

To further analyze the AL anatomy, we manually segmented individual glomeruli in the 19 male and 23 worker samples that were analyzed previously (Figures 2A and 2B). The numbers of glomeruli were sex- and species-specific, ranging from 136 to 183 glomeruli per AL (Figures 2A and 2C, two-way ANOVA: *F_sex* (1, 35) = 248, *p_sex* < 0.0001, *F_species* (5, 35) = 8.8, *p_species* < 0.0001), being consistently higher in workers (mean ± STD: 177 ± 7, *n* = 23) than males (mean ± STD:152 ± 6, *n* = 19), and strongly correlated with the overall AL volume (Figure 2D, *simple linear regression model*, *F* = 19, *p* = 0.0001, R^2^ = 0.39). We also automatically quantified glomeruli in the same smaller subset of images as discussed above. The number of automatically segmented glomeruli positively correlated with automatically calculated AL volumes (Figure 2E, *simple linear regression model*, *F* = 13.7, *p* = 0.0009, R^2^ = 0.32). The number of automatically segmented glomeruli was in agreement with manual quantification of the same samples (Figure 2F, black open circles, *simple linear regression model*, *F* = 6.3, *p* = 0.02, R^2^ = 0.17) and were sex- but not species-specific (Figure S1A, two-way ANOVA: *F_sex* (1, 25) = 4.9, *p_sex* = 0.036, *F_species* (5, 25) = 0.9, *p_species* = 0.5). Although the 32 stacks described earlier could be processed automatically, the reliability of the resulting glomerular segmentations still varied with image quality. We therefore applied the proposed predicted risk score (see STAR Methods for details) as a separate quality-control step within this automatically processed subset, which leads to retaining the 75% of images with the lowest risk scores and discarding the 25% with the highest risk scores. This risk-based filtering is distinct from the 10 of 42 stacks excluded earlier because reliable segmentation could not be obtained under the common fixed-parameter configuration. This notably improved the correlation between manual and automatic quantifications (Figure 2F, red datapoints, *n* = 24, *simple linear regression model*, *F* = 22.4, *p* < 0.0001, R^2^ = 0.5). The selected subset also retained its sex-specific distributions (Figure S1B, two-way ANOVA: *F_sex* (1, 17) = 11.8, *p_sex* = 0.003, *F_species* (5, 17) = 1.7, *p_species* = 0.18). These results indicate that strong sexual dimorphism of AL glomeruli numbers is not due to a possible human bias, as they were also observed in automatically segmented datasets. The sexual dimorphisms thus remain stable despite large evolutionary distances (over 20 Ma between *B. terrestris* and *B. pascuorum*) in bumblebees. The inconsistency in the analysis of species specificity is likely due to the relatively low number of AL samples in our sub-datasets.

**Figure 2:**
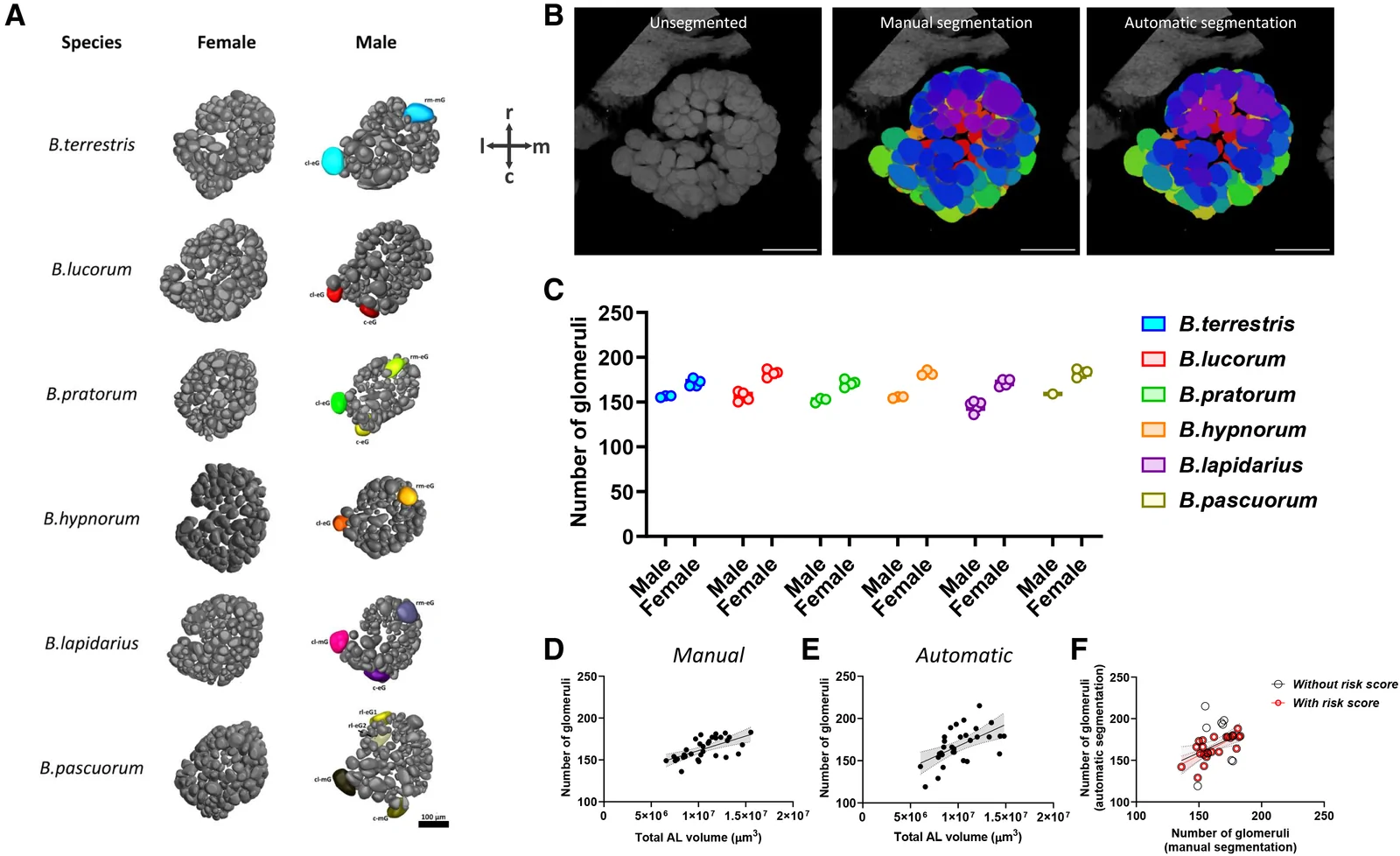
Analysis of AL glomeruli (A) Three-dimensional reconstructions of the glomeruli found in the AL of males and workers across six bumblebee species. Enlarged-/macroglomeruli are labeled in the male ALs according to their anatomical position and shown in hues of colors corresponding to previous labeling (see Figure 1). (B) Representative three-dimensional projections of unsegmented (left), manually segmented (middle), and automatically segmented (right) glomeruli within the same AL. (C) Number of glomeruli in the AL of male (M) and female (F) of *B. terrestris* (M: *n* = 3; F: *n* = 4), *B. lucorum* (M: *n* = 4; F: *n* = 4), *B. pratorum* (M: *n* = 3; F: *n* = 4), *B. hypnorum* (M: *n* = 3; F: *n* = 3), *B. lapidarius* (M: *n* = 5; F: *n* = 4), and *B. pascuorum* (M: *n* = 1; F: *n* = 4). Box-whisker plots: the box indicates 25^th^, 50^th^, and 75^th^ percentiles, the whiskers extend to min and max values. (D and E) Correlation of the total AL volume (μm^3^) and the number of glomeruli using manual segmentation (D) and automatic segmentation (E). (F) Correlation of the number of glomeruli manually segmented and the number of glomeruli automatically segmented with (in red, *n* = 24) and without (black, *n* = 32) risk score. Scale bars, 100 μm. v, ventral; m, medial; d, dorsal, l, lateral; vm, ventro-medial; vl, ventro-lateral; dl, dorso-lateral; eG, enlarged glomerulus; mG, macroglomerulus. The lines and the shaded areas (D, E, and F) represent simple linear regression lines with 95% confidence interval.

### Macroglomeruli

We next characterized the glomerular volumes (normalized by the total brain volume) across sexes and species. Unlike the workers, all male samples had several glomeruli that were dramatically larger than the rest. These macroglomeruli/enlarged glomeruli have been previously reported in the brains of male bees (although not bumblebees) and are thought to process sex pheromone information.^17^^,^^18^^,^^19^^,^^20^^,^^21^ We identified these macroglomeruli using a mathematical, but not biological functional, definition through the *K* parameter (after Bastin et al.^17^ and others; see STAR Methods for details). According to this criterion, 2–4 glomeruli were classified as macro- or enlarged glomeruli in the AL of each male sample we inspected (Figure 3). Interestingly, the positions of these glomeruli were highly similar across species, occurring in the caudal-lateral (cl-G) AL in all inspected species, and caudally (c-G) or rostral-medially (rm-G) in some, but not all, species (Figures 2A and 3). No macroglomeruli were observed in the ALs of worker bees. These results indicate that, despite previously observed (Figures 1 and 2) species differences, the overall organization of AL is well conserved across bumblebee species. Importantly, quantitative size classification and anatomical labeling were treated separately. One of the two glomeruli identified in *B. hypnorum* remained below K = 1.5 in all three male samples and therefore did not meet the quantitative definition of an enlarged glomerulus. It was nevertheless labeled as a subthreshold putative anatomical homolog because it was consistently identifiable across B. hypnorum samples and occupied the same relative antennal-lobe position as enlarged glomeruli observed in the other species (e.g., cl-G across all species and rm-G in *B. pratorum*, *B. lapidarius*, and *B. terrestris*).

**Figure 3:**
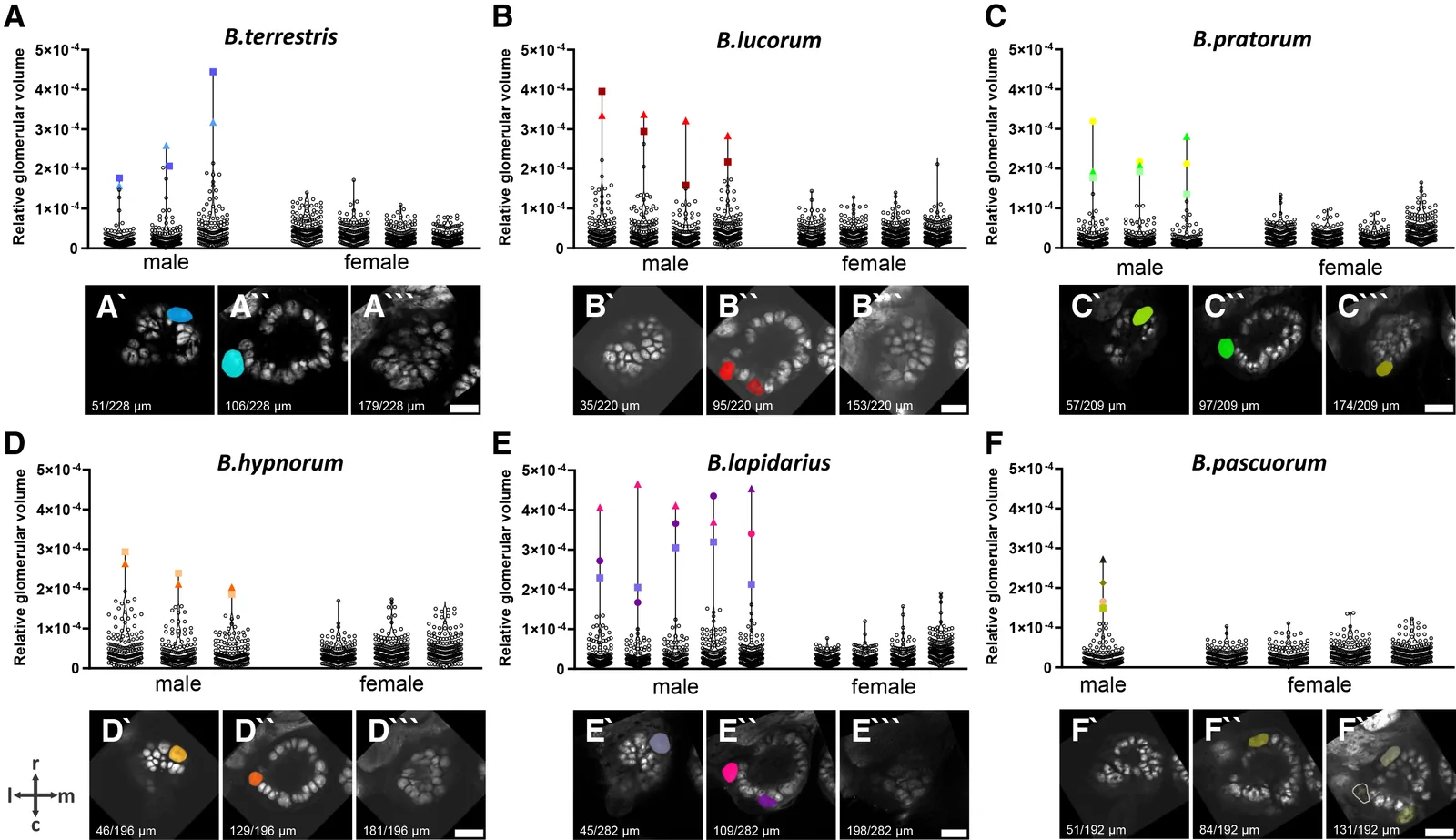
Enlarged- and macroglomeruli in the male AL of six *Bombus* species Violin plots show the distribution of glomeruli for each segmented male and female AL sample and representative confocal sections of male ALs are shown below violin plots, arranged from the ventral (left) to the dorsal (right). Macroglomeruli and enlarged glomeruli (incl. subthreshold putative macroglomeruli in *B. hypnorum*) are labeled in both the violin plots and anatomical scans with different hues of colors depending on the species: *B. terrestris* (blue, A-A```; M: *n* = 3; F: *n* = 4), *B. lucorum* (red, B-B```; M: *n* = 4; F: *n* = 4), *B. pratorum* (green, C-C```; M: *n* = 3; F: *n* = 4), *B. hypnorum* (orange, D-D```; M: *n* = 3; F: *n* = 3), *B. lapidarius* (purple, E-E```; M: *n* = 5; F: *n* = 4), and *B. pascuorum* (yellow, F-F```; M: *n* = 1; F: *n* = 4). Shapes within the violin plot further indicate the order of depth they were found in from dorsal to ventral: square-triangle-circle-diamond. Each confocal section is labeled with the depth of picture shown relative to the overall depth of the confocal stack. Scale bars, 100 μm. v, ventral; m, medial; d, dorsal, l, lateral.

### Olfactory sensilla and foraging patterns

Glomeruli consist of synaptic connections between olfactory receptor neurons, projection neurons, and interneurons. We thus hypothesized that larger AL volumes may be correlated with the higher number of receptor neurons, and by extension—higher density of antennal olfactory sensilla. In addition, the number of glomeruli is likely to reflect the diversity of olfactory receptors expressed, and as such may also correlate with the number of olfactory sensilla. We thus asked whether the observed sexual dimorphisms in the antennal lobe are reflected by the density of olfactory sensilla on the bees’ antennae (Figures 4A and 4B). We used scanning electron microscopy (SEM) to image pre-distal antennal flagellomeres of bees, analyzing one antenna per bee. We identified placoid (SP), basiconic (SB), and trichoid (ST) sensilla by their visual appearance. The SPs have a flat appearance, are elongated and rich in pores. The SBs are hair-like, short and thick-pored sensilla with a grooved surface, and are generally not curved. The STs are also hair-shaped but they are thinner and longer than SBs, are often curved and may have an apical pore. The density of the main olfactory (placoid and basiconic) sensilla was sex- but not species-specific (Figure 4C, two-way ANOVA: *F_sex* (1, 38) = 11.5, *p_sex* = 0.002, *F_species* (5, 38) = 0.98, *p_species* = 0.44), being consistently higher in workers (mean ± STD: 36.3 ± 6.3, *n* = 23) than in males (mean ± STD: 30.7 ± 4.1, *n* = 22). Basiconic sensilla were found exclusively in females. The density of placoid and basiconic sensilla had a strong positive correlation with the overall number of glomeruli, calculated at species/sex level (Figure 4D, *simple linear regression model*, *F* = 24.8, *p* < 0.0001, R^2^ = 0.37), indicating that the increase in glomerular numbers may be accompanied by new subtypes of olfactory sensilla that house neurons expressing olfactory receptors, unique for that sex or species. However, there was no significant correlation between the overall AL volume and sensillar density (Figure S1C, *simple linear regression model*, *n* = 42, *F* = 2.6, *p* = 0.12, R^2^ = 0.06). This result implies that the larger AL volume primarily reflects an expansion of local interneurons and second-order neurons, rather than an increased number of antennal receptor neurons. The density of trichoid sensilla, which mediate mechanoreception and chemoreception, was significantly different between species but not between sexes (ANOVA: *F_sex* (1, 38) = 0.08, *p_sex* = 0.78, *F_species* (5, 38) = 2.9, *p_species* = 0.02).

**Figure 4:**
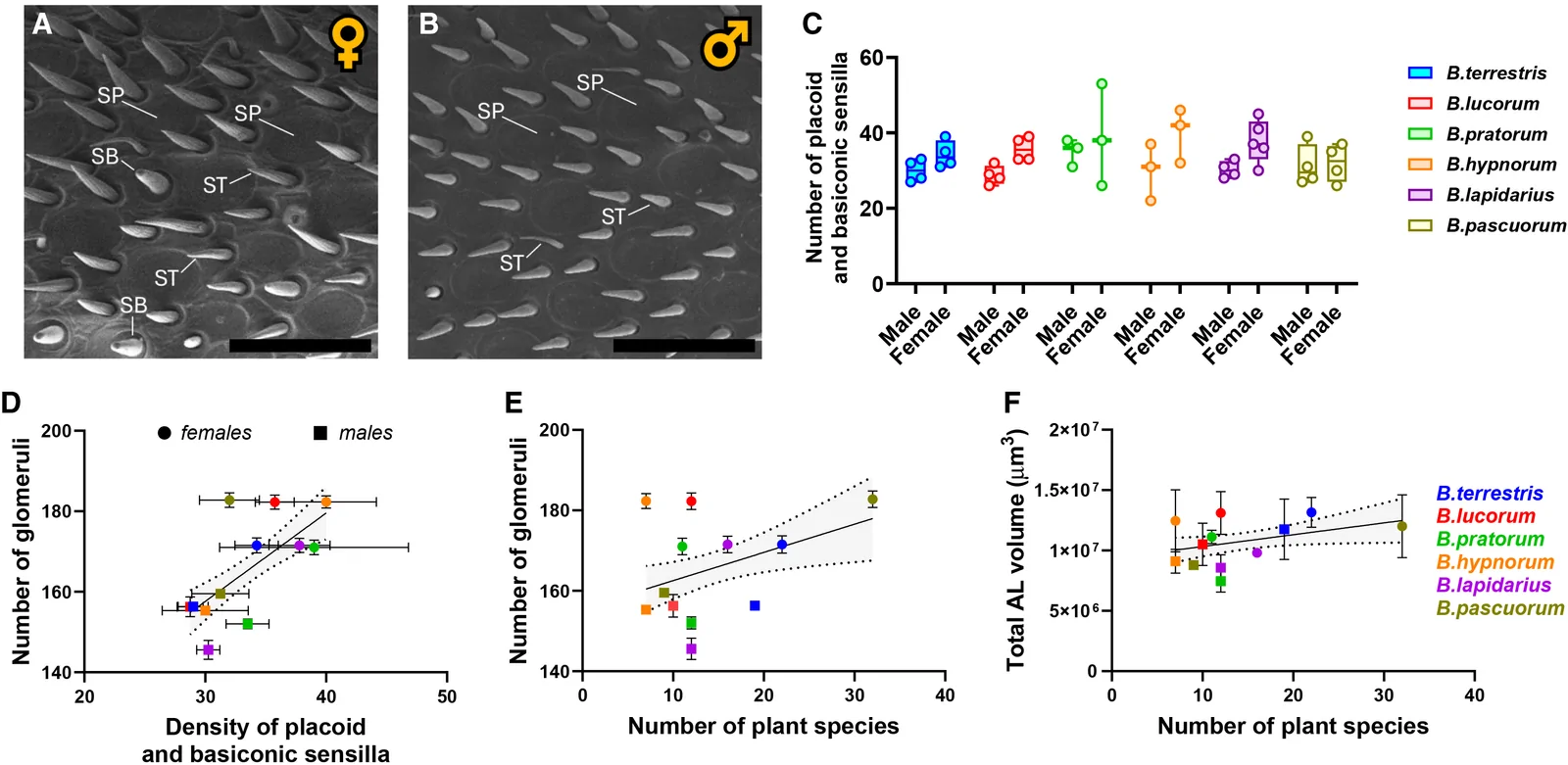
Olfactory sensilla and foraging repertoires (A and B) Scanning electron microscopy (SEM) representative images showing the types of sensilla present on the antenna of a *Bombus hypnorum* female (A) and male (B). SB, basiconic sensilla; ST, trichoid sensilla; SP, placoid sensilla. Scale bars, 40 μm. (C) The number of olfactory (placoid and basiconic) sensilla on the antenna of males (M) and females (F) of *B. terrestris* (M: *n* = 4; F: *n* = 4), *B. lucorum* (M: *n* = 4; F: *n* = 4), *B. pratorum* (M: *n* = 3; F: *n* = 3), *B. hypnorum* (M: *n* = 3; F: *n* = 3), *B. lapidarius* (M: *n* = 4; F: *n* = 5), and *B. pascuorum* (M: *n* = 4; F: *n* = 4). Box-whisker plots (C, D, F, and I): the box indicates 25^th^, 50^th^, and 75^th^ percentiles, the whiskers extend to min and max values. (D) Correlation of the number of glomeruli and the density of olfactory sensilla. Each data point on the graph corresponds to male or female bees of one species. (E) Correlation of the number of glomeruli and the number of plant species visited by males and workers of the six *Bombus* species. (F) Correlation of the total antennal lobe volume and the number of plant species visited by males and workers of the six *Bombus* species. The lines and the shaded areas represent simple linear regression lines with 95% confidence interval (D–F). Error bars represent standard deviations.

Finally, we asked whether anatomical dimorphisms that we observed have functional implications and correlate with the diversity of plants that the bees foraged on. For each sex and species of bees, we calculated the number of plant species that these bees were collected from in this study (see Table S1). The number of plant species ranged from 7 for both sexes of *B. hypnorum* to 32 for *B. pascuorum* workers. Interestingly, we observed several unique plant-bee pairs at the sex and species level (e.g., *B. lapidarius* workers—*Cardamine pratensis*; *B. pascuorum* males and workers—*Ballota nigra* and *Vicia cracca*; *B. pratorum* workers—*Campanula latifolia*; *B. terrestris* males and workers—*Buddleja davidii*, etc.), although these were not correlated with the AL morphology in an obvious way. We found that the number of visited plant species has a strong positive correlation with the number of AL glomeruli (Figure 4E, *simple linear regression model*, *n* = 42, *F* = 6.5, *p* = 0.015, R^2^ = 0.137) and the AL volume (Figure 4F, *simple linear regression model*, *n* = 42, *F* = 4.1, *p* = 0.049, R^2^ = 0.1). These results indicate that broader foraging repertoires may at least partially rely on perception and discrimination of a higher number of floral volatiles, which in turn require higher olfactory sensitivity and specificity.

## Discussion

This study has been motivated by the apparent lack of knowledge about bumblebee neuroanatomy and neuronal basis of behavior, outside of the workers of commercially available species—*B. terrestris*, *B. impatiens*, and *B. ignitus*. We analyzed wild-caught sympatrically occurring bumblebees of 6 species and found that, while there are no obvious sex or species dimorphisms of their overall brain sizes, their antennal lobes are dramatically sexually dimorphic. Worker bumblebees have larger antennal lobes, more glomeruli, and more olfactory sensilla, compared with males. Males, however, have 2–4 macroglomeruli that occur in evolutionary conserved locations. No worker bees have macroglomeruli. Finally, we observed that bees that visit a higher diversity of plants tend to have larger antennal lobes with more glomeruli.

The overall brain volumes, as well as absolute and relative AL volumes and the numbers of AL glomeruli have a range of variability for each sex and species analyzed. The variation in absolute brain sizes is not surprising, given that the overall body size of the bee may differ due to its nutrition during development, genetic makeup and other factors. While the number of AL glomeruli appears to be very consistent in some insects (e.g., *Drosophila*^22^^,^^23^), is more variable in others (mosquitoes,^24^^,^^25^^,^^26^ bees,^20^^,^^27^ butterflies,^28^ and ants^29^). Hence, these differences are likely to be genuine and due to factors that are yet to be characterized. However, it is also possible that the differences, as least partially, are due to human errors during manual segmentation of suboptimal quality images.

Sex-specific glomerular organization, i.e., the lower numbers of glomeruli in males, is consistently observed across Hymenopteran species and, in bees, has been previously reported in *Apis mellifera*,^27^ *Melipona scutellaris*,^20^ *Eucera berlandi*,^21^ and *Euglossa dilemma*.^30^ The number of glomeruli across species is likely to correspond to the number of olfactory receptor (OR) genes in their genome.^31^ Although the genomes for most of the species in this study have been sequenced,^32^^,^^33^^,^^34^ only the ORs for *B. terrestris* have been extensively analyzed. The *B. terrestris* genome contains 164 OR genes^34^ and this report identified ∼170 glomeruli in *B. terrestris* females (similar to the ∼160 glomeruli previously found in *B. terrestris*^7^). The counted glomeruli in *B. terrestris*, therefore, not only correspond with previous examinations of the AL but are also similar to the number of ORs identified in the genome.

We have observed higher density of the main olfactory (placoid and basiconic) sensilla on the penultimate flagellomere of worker bees, compared to males. Higher numbers of placoid sensilla are associated with enhanced olfactory acuity in *B*. *terrestris*^35^ and were hypothesized to facilitate detection of female pheromones in honeybees.^36^^,^^37^ Furthermore, we observed sensilla basiconica exclusively in worker bees. Neurons that innervate these sensilla are likely to account for the additional glomeruli that are present in workers, but not in males. In contrast, trichoid sensilla had similar densities in females and males, reflecting their comparatively weaker involvement in odor perception. Future studies should attempt to elucidate the function of the subtypes of trichoid sensilla. It is possible that at least one of the subtypes may differ between sexes. We observed that the density of trichoid sensilla differed between species. However, inspecting the behavioral ecology traits of our study species, we found no obvious links with nesting behavior, colony size, or floral preferences, but this may be due by the fact that the different sub-types of trichoid sensilla could not be analyzed separately here.

To date, this is the first analysis of macroglomeruli in any *Bombus* species. Macroglomeruli (referred to here as enlarged glomeruli) are implicated in species-specific sex pheromone detection^27^^,^^38^^,^^39^ and were found to be highly diverse across the AL of different male bumblebee species. Despite this diversity, their anatomical organization across species appears similar, with minimal regard for phylogenetic relatedness. Notably, one enlarged glomerulus—cl-G—was consistently observed in the same location across all analyzed bumblebee species, suggesting a conservation of this glomerulus across genera, spanning at least 25 million years of evolution.^14^^,^^40^ Previously, a conserved macroglomerulus has also been observed in the AL of males across several *Apis* species.^17^ In *Apis mellifera*, this macroglomerulus exhibits a specific response to the queen sex pheromone 9-ODA.^39^ It is thus likely that cl-G is innervated by neurons that respond to a conserved bumblebee pheromone.

Methodologically, there are three main innovations in this study. First, we collected and used wild bees, which is not a usual practice in insect neuroscience research, where laboratory-bred animals from inbred colonies are normally used. This approach allowed us to study bumblebee species that are not commercially available or easy to keep in the laboratory (*B. pascuorum*, *B. pratorum*, *B. lapidarius*, *B. hypnorum*, and *B. lucorum*), and to draw more general conclusions, without the risk that our findings are artifacts of inbreeding. To the best of our knowledge, this is the first study that characterized brain anatomy of *B. pascuorum*, *B. pratorum*, *B. lapidarius*, *B. hypnorum*, and *B. lucorum*. We also collected the information about foraging choices of the bees, a subset of which we analyzed here. However, because the bees were field collected, we were unable to control for their age, developmental conditions, feeding status or previous foraging experiences. These factors are likely to introduce additional variability into our data. The possible influence of these factors on bumblebee brain development, in general, and olfactory system, in particular, may be investigated further by using laboratory or field-collected bumblebee colonies under controlled laboratory conditions. Studies of solitary (cuckoo) bumblebees and foraging specialists (*B. consobrinus* and *B. gerstaeckeri*) would also be very informative, to investigate the effects of sociality and foraging repertoire on olfactory system.

Second, we analyzed anatomy of both the peripheral (antennal sensilla) and the central (brain) olfactory systems. Previous studies on bumblebees and many other non-model Hymenoptera focused either on olfactory sensilla and the relationship of their density to variation in life-history traits (e.g., pollen diet specialization in bees and wasps,^41^ variation in social behavior in bees^42^), or on the brain anatomy (e.g., sexual dimorphism in solitary bee,^43^ caste dimorphism in social wasps,^44^^,^^45^ variation in social behavior in wasps,^46^ effect of social parasitism in wasps,^47^ effect of colony size in ants,^48^ etc), but rarely included both.

Third, we developed a pipeline for automatic segmentation of glomeruli from confocal images. This pipeline eliminates the most time-consuming step in the data analysis—manual segmentation of confocal images—which normally took us around 5–8 h per antennal lobe and instead automatically processes each antennal lobe in 102.57 s on average when running on an Intel Core i9-10920X CPU @ 3.50 GHz.

The method is based on a 3D watershed formulation and is designed to identify well-defined, morphologically consistent glomerular structures without sample-specific intervention. Under the fixed-parameter unattended configuration evaluated here, 32 of 42 image stacks (76%) fell within the operating range of the pipeline and could be processed successfully. The principal operational boundary was encountered when weak or spatially heterogeneous contrast and elevated image noise compromised foreground or marker extraction before watershed segmentation. Within the successfully processed images, the quantitative risk score provides a separate quality-control mechanism for identifying lower-confidence segmentations prior to downstream analysis.

### Limitations of the study

The main limitation of our study is the low number of samples analyzed for each sex and species. This reduces the statistical power of the inter-species comparisons and robustness of our conclusions. In addition, we are unable to tell whether the intra-specific variability in the glomeruli count is a genuine phenomenon or an artifact. All samples in this study were field-collected, and thus were not controlled for age, developmental conditions, feeding status or previous foraging experiences, that may have a genuine effect on their antennal lobe organization and are likely to introduce additional variability into our data, reducing the statistical power of the comparisons conducted and robustness of the conclusions.

Another possible limitation of our study is that the antennal and the brain samples were harvested from different individual bees, which, nevertheless, were all derived from the same overall sample set collected around Durham, UK. We thus cannot correlate antennal and brain quantifications at an individual level, and can only compare the averages for each sex and species.

The primary limitation of our automatic segmentation method is its dependence on sufficiently stable foreground/background separation and local boundary information when operated with a single common parameter set. Strong spatial variation in signal-to-background contrast, weak intensity gradients at interglomerular boundaries, or elevated high-frequency noise can compromise pre-watershed foreground or marker extraction, with these upstream errors propagating to incomplete, unstable, or over-segmented outputs. It is important to note that these cases remained amenable to expert manual annotation because an annotator could integrate three-dimensional morphology across successive optical sections and orthogonal views. Addressing these cases automatically would instead require sample-specific parameter adjustment and manual quality control, which was deliberately excluded from the fully automated workflow evaluated here. Future work could extend the operating range through adaptive foreground extraction, noise-aware marker generation, or automatic per-volume parameter estimation.

## Resource availability

### Lead contact

Requests for further information and resources should be directed to and will be fulfilled by the lead contact, Olena Riabinina (olena.riabinina@durham.ac.uk).

### Materials availability

This study did not generate new unique reagents.

### Data and code availability

The code for custom 3D watershed segmentation and risk-scoring pipeline is available in an open GitHub repository: https://github.com/Tom-E-Durham/bombus-antennal-lobe; https://doi.org/10.5281/zenodo.21294364; version v1.0.0. All other data reported in the manuscript will be shared by the lead contact upon request.

## Acknowledgments

We thank Esme Padgett, Serena Broad, Lottie Apsey, and Hannah Cowling for assistance with field collections and bee barcoding, Stefania Crespi and Giorgia Nicolucci for assistance with SEM image acquisition, Prof. Wayne Dawson for plant identifications, and Dr. Vivek Nityananda for providing *B. terrestris* specimens for preliminary studies. This study was supported by Durham Seedcorn grant (WT 2870506 to O.R.). J.O. was funded by the Erasmus+ program for his placement at Durham University during this project. WCGR was funded by Durham Doctoral Scholarship. IJM was funded by NLD BBSRC DTP (10.13039/501100000268Biotechnology and Biological Sciences Research Council grant BB/T008695/1). The authors gratefully acknowledge the Durham Biosciences Bioimaging Facility for their support and assistance in this work.

## Author contributions

Conceptualization, J.O., W.E., S.C., A.A.-A., C.P., and O.R.; methodology, J.O., W.E., W.C.G.-R., I.M., and T.W.; investigation, J.O., W.E., W.C.G.-R., I.M., T.W., M.B., and S.C.; formal analysis, J.O., W.E., and W.C.G.-R.; funding acquisition, J.O., W.C.G.-R., I.M., A.A.-A., C.P., and O.R.; project administration, O.R.; writing – original draft, J.O. and W.E.; writing – review and editing, all co-authors.

## Declaration of interests

The authors declare no competing interests.

## STAR★METHODS

### Key resources table

**Table:** 

REAGENT or RESOURCE	SOURCE	IDENTIFIER
**Antibodies**		

mouse anti-SYNORF1	DSHB	CAT#3C11; RRID: AB_528479
Cy3-conjugated Goat (anti-mouse)	Jackson ImmunoResearch	CAT#115–165–062; RRID: AB_2338685

**Biological samples**		

Mounted SEM samples of bumblebee antennae	This study	

**Chemicals, peptides, and recombinant proteins**		

Ethanol, 100%	Fisher Chemicals	16606002; CAS: 64-17-5
PBS (10X), pH 7.4	GibcoTM	CAT#70011036
TRIS, 1.0M buffer soln., pH 7.4	Thermo Scientific	AAJ60202K2; CAS: 77-86-1
Dimethyl sulfoxide (DMSO)	New England Biolabs	B0515; CAS: 67-68-5
Normal goat serum (NGS)	Jackson ImmunoResearch	CAT#005-000-121; RRID: AB_2336990
Methanol	Fisher Chemicals	423955000; CAS: 67-56-1
Methyl salicylate	Alfa Aesar	11408800; CAS: 119-36-8
Exaktosil N21A	Bredent	5400116A
Exaktosil N21B	Bredent	5400116B

**Critical commercial assays**		

DNeasy® Blood and Tissue Kit	Qiagen	CAT#69506
NucleoSpin Gel and PCR Clean-up	Macherey-Nagel	CAT#740609.50

**Experimental models: Organisms/strains**		

Bombus terrestris	N/A	N/A
Bombus pascuorum	N/A	N/A
Bombus pratorum	N/A	N/A
Bombus lapidarius	N/A	N/A
Bombus lucorum	N/A	N/A
Bombus hypnorum	N/A	N/A

**Oligonucleotides**		

LepF	Hebert et al.^49^	N/A
LepR	Hebert et al.^49^	N/A
HCO-Hymeno-2	Villalta et al.^50^	N/A
HCO-Hymeno-1	Villalta et al.^50^	N/A
16SWb	Williams et al.^51^	N/A
16SIR	Williams et al.^51^	N/A

**Software and algorithms**		

Unipro UGENE v. 42.0	UGENE	RRID: SCR_005579
NCBI BLAST®	NCBI	RRID: SCR_001598
Zen Blue software (V2.6)	Zeiss	RRID: SCR_013672
ImageJ (v1.54p)	NIH	RRID: SCR_003070
Dragonfly 3D world	Dragonfly	RRID: SCR_025150
MultiStackReg	Thevenaz et al.^52^	RRID: SCR_016098
The Hymenoptera Anatomy Ontology (HAO) portal	Yoder et al.^53^ (http://glossary.hymao.org/projects/32/public/ontology/)	RRID: SCR_003340
Custom 3D watershed segmentation and risk-scoring pipeline	This study	https://github.com/Tom-E-Durham/bombus-antennal-lobe; https://doi.org/10.5281/zenodo.21294364; version v1.0.0
Python 3.8.15	Python	RRID: SCR_008394
GraphPad Prism version 10.2.0		

**Other**		

Stainless Steel Beads, 2.4 mm	GENIE	SI-BS02

### Experimental model and study participant details

#### Bumblebee collection and identification via DNA barcoding

Wild worker and male bumblebees of *B. terrestris*, *B. pascuorum*, *B. pratorum*, *B. lapidarius*, *B. hypnorum*, and *B. lucorum* were collected while resting on a flower from multiple locations across County Durham, UK, during two time periods: 06 June – 12 August 2022 and 21 June – 09 August 2023. Collection permits were not required for this work. The age of bumblebees remained unknown. Bumblebees were captured from flowers using Falcon 15 mL Conical Centrifuge Tubes, placed in a cooler (Coleman 5QT Performance Thermal Box | 4.7 L Capacity Cooler) and transported to the laboratory, where they were sexed by observing a sting/genitalia under a dissection microscope, and stored in a freezer at −20 °C until further analysis. Samples that were used for antennal analysis were stored in 100% ethanol (Fisher Chemicals, 16606002), and samples for brain anatomy analysis were frozen dry for 10–12 months.

### Method details

#### Bumblebee identification via DNA barcoding

DNA was extracted from the middle leg of each using the DNeasy Blood and Tissue Kit (Qiagen, Hilden, Germany, #69506). The leg was vortexed for 10 min in 400 μL of PBS (GibcoTM, #70011–036) with approximately 5g of metal beads (GENIE, Stainless Steel Beads, 2.4 mm, #SI-BS02) in a 1.5 mL Eppendorf tube to homogenise the sample. After this, the manufacturer’s instructions were followed. The extracted DNA was used as a template in PCR reactions using LepF/LepR,^49^ HCO-Hymeno-2/Hymeno-1^50^ and 16SWb/16SIR^51^ primer pairs to amplify Cytochrome *c* Oxidase I (COI) and/or 16S rRNA mitochondrial genes. Following successful amplification, the DNA fragments were purified using NucleoSpin Gel and PCR Clean-up (Macherey-Nagel, #740609.50) according to the manufacturer’s instructions. The purified PCR products were sent to GENEWIZ UK Ltd (Essex, UK) for Sanger Sequencing with the forward or reverse primers used in the PCR reaction. Sequencing results were visualised using Unipro UGENE v. 42.0. High-quality sequences were run through NCBI BLAST to identify the bee species.

#### Plant identification

The majority of the plants that the bees were collected from were identified using plant morphology keys,^54^ based on morphological features observed in the field. To ensure accuracy, each plant was photographed in the field to document its morphological characteristics. The images were also later shared with Professor Wayne Dawson, an expert in plant identification, who provided precise identifications for each specimen.

#### Brain dissection, immunostaining, clearing and mounting

The protocol for immunostaining bumblebee brains using zinc–formaldehyde (ZnFA) fixation was adapted from Rother et al. (2021)^8^ and Ott (2008).^55^ In short, dry frozen bumblebees were decapitated and the heads were plunged in ice-cold HEPES-buffered saline (HBS; 150 mM NaCl, 5 mM KCl, 5 mM CaCl_2_·6H_2_O, 25 mM sucrose, and 10 mM HEPES).^55^ Mandibles, proboscis, antennae, and dorsal exoskeleton were removed, and the heads were immediately transferred to ZnFA (18.4 mM ZnCl_2_, 135 mM NaCl, 35 mM sucrose, and 1% formaldehyde) for fixation. Bumblebee heads were incubated in ZnFA for 20–24 h at room temperature (RT). The following day, the brains were moved to ice-cold HBS, fully dissected, rinsed twice with fresh HBS, and kept in HBS overnight at 4 °C. Brains were then washed in 0.1 M Tris buffer, pH 7.4 (Thermo Scientific; #77–86–1) (3 × 10 min), permeabilized in a Dimethyl sulfoxide (DMSO; New England Biolabs; #B0515B0151A) and Methanol (Fisher Chemicals, #67–56–1) mixture (20:80; 85–90 min), and washed again in 0.1 M Tris buffer (3 × 10 min) at RT. Brains were blocked in 5% Normal goat serum (NGS; Jackson ImmunoResearch Labs, AB_2336990) in PBT for 1–2 h at RT, then incubated with the primary antibody, anti-SYNORF1/3c11 (1:100; DSHB, AB_528479), in 5% NGS in PBT for 3–5 days at 4°C. After incubation, brains were washed in PBT (8 × 30 min), incubated with a Cy3-conjugated Goat anti-mouse secondary antibody (1:100; Jackson ImmunoResearch Labs, #AB_2338685) in 5% NGS in PBT for 2–4 days at 4 °C, and again washed in PBT (8 × 30 min).

Synapsin-stained bumblebee brains were cleared as described by Rother et al. (2021).^8^ Brains were dehydrated in an ascending ethanol series (30%, 50%, 70%, 90%, 95%, 2 × 100%; 15 min each) in deionised water. Approximately half of the ethanol was then replaced with pure methyl salicylate (Alfa Aesar, #119–36–8) and the tubes were left undisturbed at room temperature (RT). When the brains had sunk to the bottom of the tube (∼30 min), the residual ethanol was replaced with methyl salicylate, and the samples were stored upright at 4 °C.

Mounting chambers were created by stacking 12 Reinforcement Ring Labels Stickers (13 mm in diameter; TownStix), resulting in a chamber with a height of approximately 0.80 mm. Brains were mounted individually in these chambers using methyl salicylate, covered with a 13 mm round coverslip, and sealed with a 50/50 mixture of Exaktosil N21A and Exaktosil N21B (Bredent #5400116A and #5400116B). Mounted samples were imaged within 3–4 h of sealing.

#### Confocal microscopy

All mounted samples were imaged using a Zeiss 800 laser scanning confocal microscope (LSCM) using 561 nm excitation with an emission detection window of 400–623 nm. Images were acquired using ZEN Blue software (version 2.6). Whole brains were imaged as tiles at a 512 × 512 pixel frame size and 16-bit depth, using an EC Plan-Neofluar 10x/0.3 M27 air objective (Zeiss #420340–9901–000) with a digital zoom of 0.5 and a z-step size of 5.27 μm (dimensions: x: 2428.32 μm, y: 2428.32 μm). Antennal lobes were imaged at a 512 × 512 pixel frame size and 16-bit depth using a Plan-Apochromat 20x/0.8 M27 air objective (Zeiss #420650–9903–000) with a digital zoom of 0.7 and a z-step size of 0.62 μm (dimensions: x: 456.36 μm, y: 456.36 μm). All Z-stacks were corrected for scanning depth by adjusting the laser intensity during image acquisition. Images were exported as TIF and CZI files from Zeiss ZEN Blue. Whole brain tile images were imported into Fiji as a single stack, separated into tile stacks using the “Deinterleave” command, and individual tiles were saved as TIF files. The “Grid/Collection Stitching” command in ImageJ was then used to stitch the tile sequences. Antennal lobe image sequences were imported into *Fiji* and saved as a TIF sequence.

#### 3D segmentation of the antennal lobe and whole brain

All segmentations were performed in Dragonfly 3D world (Object Research Systems, Canada) using TIF Z-stacks. The x, y, and z dimensions (μm) were extracted from the metadata of the original CZI files using the “Properties” command in *Fiji* and entered into the “Spacing (μm)” option upon importing the data to Dragonfly. Variations in brain size caused some samples to float inside the chamber, resulting in movement during imaging of the antennal lobes. These shifts were corrected using the “Rigid Body” setting in the StackReg *Fiji* plugin.^52^

The antennal lobes were segmented in the “XY view” using the “Segment” menu in Dragonfly 3D world. For each glomerulus, a separate Region of Interest (ROI) layer was created by selecting the image layer in “Geometry” under ROI Tools > Basic > New. The “Freehand Region for ROI” tool in ROI Painter > 2D View Tools was used to outline the beginning, middle, and end of each glomerulus; additional outlines were added if the glomerulus morphology was not round. Interpolation was then applied under ROI Tools > Operations > Interpolate (Z) to complete the segmentation of the glomerulus between the given outlines. To reduce the graphical load, after approximately 20 glomeruli were segmented, all single ROI layers were selected and merged into a multi-ROI by right-clicking and selecting “Create Multi-ROI from ROIs.” Single ROIs were then deleted. After segmenting the entire antennal lobe, all multi-ROI layers were combined using the Boolean operation “Union.” Glomeruli counts were extracted from the final multi-ROI using Statistical Properties > Labeled Class Count. Individual glomeruli volumes were obtained by right-clicking on the multi-ROI layers, selecting Connected Components > Analysis. In the Object Analysis menu, the volume outputs were included under “Select Statistical Properties for Analysis.” The Volume column was selected, and scalar values were extracted from Classes and Scalar Information > Export Scalar Values. This process provided the volume of each glomerulus and the total number of glomeruli for each segmented antennal lobe. Only one antennal lobe was segmented per bee, with no preference for lateralisation. Due to the thickness of the samples and the limited working distance of the 20x objective (0.55 mm), the morphology of the antennal lobe was occasionally distorted by the objective touching the coverslip. These samples were excluded from volume measurements but included in the count of the total number of glomeruli.

The volume of the whole brain was determined **i**n Dragonfly 3D World. An ROI layer was created under ROI Tools > Basic > New, and the entire brain was then segmented by thresholding using ROI Tools > Range. The threshold range that encompassed the whole brain was selected in the histogram window and added to the ROI. To reduce noise, the ROI was processed with Morphological Operators (ROI Tools > Morphological Operations): Close and Smooth (Dimensionality: 3D; Shape: Cube; Range: Range; Kernel Size: 3). The view was then switched to 3D, and the “Freehand Region for ROI” tool under ROI Painter > 3D View Tools was used to clear any remaining noise or unwanted selections. The volume of the segmented brain was then extracted from Statistical Properties > Volume (Labeled Voxels).

#### Automatic watershed segmentation of antennal lobe glomeruli

A marker-controlled 3D watershed framework^56^^,^^57^ was applied to segment confocal volumes into anatomically meaningful instances. The pipeline consisted of five sequential stages: preprocessing, binary masking, distance transform, marker extraction, elevation map construction and final watershed segmentation.

The full implementation, environment setup, package versions, version-controlled configuration files and a runnable example are archived at https://doi.org/10.5281/zenodo.21294364 (release v1.0.0; source repository: https://github.com/Tom-E-Durham/bombus-antennal-lobe). The workflow produces instance label maps, per-glomerulus measurements, per-sample parameter records and risk-score outputs from exported TIF image stacks.

##### Preprocessing

The input data were 3D intensity volumes *I*(*x*,*y*,*z*) acquired from confocal microscopy, typically with a resolution of 512 × 512 × Z. To minimise inter-sample intensity variation, we first performed global *Z* score standardisation:

$$I^{\prime }=\left(I-\mu \right)/\sigma$$

where μ and σ denote the global mean and standard deviation computed across all samples. Subsequently, a min–max normalisation rescaled the standardised volume to the range [0,1]:

$$I_{norm}=\left(I^{\prime }-\min\left(I^{\prime }\right)\right)/\left(\max\left(I^{\prime }\right)-\min\left(I^{\prime }\right)\right)$$

To enhance local structural contrast, we applied a percentile-based stretch:

$$I_{ce}=clip\left(\left(I_{norm}-v_{low}\right)/\left(v_{high}-v_{low}\right),0,1\right)$$

where *v*_*low*_, *v*_*high*_ correspond to percentiles *p*_*low*_, *p*_*high*_. This step suppressed extreme outliers and accentuated mid-to-high intensity regions relevant to anatomical boundaries. A 3D Gaussian filter with a small standard deviation *σ*(in voxel units) was then applied to suppress high-frequency noise while preserving fine boundaries. The resulting smoothed volume is denoted as *I*_*d*_, which serves as input to the subsequent segmentation stage.

##### Binary mask and distance transform

Binary segmentation converted the pre-processed volume into a coherent foreground support domain. We employed Otsu’s global thresholding method, which selects a threshold *T* that maximises the between-class variance:

$$\sigma_{b}^{2}\left(T\right)=w_{0}\left(T\right){\left[\mu_{0}\left(T\right)-\mu_{T}\right]}^{2}+w_{1}\left(T\right){\left[\mu_{1}\left(T\right)-\mu_{T}\right]}^{2}\text{,}$$

where *w*_0_,*w*_1_ are class probabilities, *μ*_0_,*μ*_1_ are the class means below and above *T*, and *μ*_*T*_ is the global mean intensity. The optimal threshold *T*_*Otsu*_ was scaled by a factor *threshold_factor* ∈ (0,1] to adapt to weakly contrasted regions:

$$Mask_{raw}\left(x\right)=I_{d}\left(x\right)>threshold\;factor\times T_{Otsu}$$

Setting *threshold_factor* < 1 increased foreground coverage, reducing missed detections in low-contrast volumes. A morphological closing operation using a spherical structuring element ball(2) filled small holes and bridged narrow gaps, yielding a contiguous foreground mask M that constrains subsequent morphology and watershed operations.

Given the foreground mask M, we computed the 3D Euclidean distance transform:

D(x)=min||x−y||,wherey∈background.

To enhance separability between object centers, the distance map was raised to a power:

$$D_{p}=D^{\gamma },\gamma =2.5\text{.}$$

##### Marker extraction

Marker generation identified discrete seeds representing potential object centers for the watershed algorithm. We first estimated a robust threshold *P*_*med*_ by taking a chosen percentile (60%) of the positive values of *D*_*p*_, and set all voxels below *P*_*med*_ to zero, producing a filtered map. This suppresses weak responses near boundaries and thin connecting necks. Within *D*_*f*_, shallow valleys are further weakened by a suppression factor *s*∈(0,1), applied to voxels with 0<*D*_*f*_<*V*, where *V* is a lower percentile threshold. This “valley suppression” step prevents artificial bridges between neighboring peaks. Local maxima are then detected on the suppressed map using a minimum inter-peak distance *d*_*min*_ (in voxels) and a small spherical neighborhood of radius *r*. Peak detection is constrained by the foreground mask *M* to ensure all seeds lie within valid tissue regions. The resulting peaks {*p*_*i*_} are rasterised into a sparse binary volume and labeled via connected-component analysis to form an integer marker map *K*, in which each label corresponds to one seed.

##### Composite elevation map and watershed segmentation

To guide the watershed flooding, we construct a composite elevation map that combines intensity gradient and distance information. Both the gradient magnitude *G* (computed on *I*_*d*_) and the power-enhanced distance transform *D*_*p*_ are normalised to [0,1], yielding *G*_*norm*_ and *D*_*norm*_. The final elevation map is defined as:

$$E=\left(1-\alpha \right)G_{norm}+\alpha \left(1-D_{norm}\right)$$

where *α* ∈ [0,1] balances the contributions of edge precision and shape regularity. A subsequent power enhancement *E*′ = *E*^1.3^ increases the contrast between basins and ridges, sharpening object boundaries. The elevation map thus acts as an energy surface for watershed expansion, where low-potential basins correspond to object interiors and high-potential ridges delineate boundaries.

Traditional marker-controlled watershed segmentation commonly relies on either the distance transform or the image gradient as the energy surface. Distance-based flooding provides effective object separation but often leaks across weak boundaries, whereas gradient-based flooding is sensitive to local noise and intensity inhomogeneity. To overcome these limitations, we employ a composite elevation map *E*′ that integrates both distance and gradient information to produce a smoother and anatomically consistent energy landscape for flooding. The final segmentation is obtained by applying a marker-controlled 3D watershed on the elevation map *E*′, using the marker map *K* and the foreground mask *M*. The algorithm follows the principle of priority flooding: each labeled seed initiates a basin at a local minimum, voxels are assigned in order of increasing elevation, and boundaries emerge where expanding basins meet. Propagation is constrained by *M*, ensuring all labels remain confined within anatomically valid regions. After flooding, small or spurious fragments are removed via size-based regularisation: objects with fewer than *N*_*min* voxels are discarded and relabeled as background. This post-processing acts as a statistical regulariser, assuming true anatomical units exceed a minimal volume. The resulting label map provides distinct, non-overlapping 3D instances, and the overall framework yields a reproducible, parameter-controlled segmentation approach that balances boundary precision and structural completeness under moderate shape variability and imperfect intensity contrast.

#### Risk scoring and segmentation quality control

To enable risk-aware pre-screening of the dataset, we computed two complementary risk scores from frequency-domain statistics of the raw projections: a dataset-level global risk score (*risk*_*global*_) and a learned predicted risk score (*risk*_*pred*_). High-risk samples are prioritised for manual inspection, while low-risk samples are processed by our watershed algorithm pipeline, improving overall reliability under a limited annotation budget. For each piece of data, we analyzed a set of rendered 2D projections and extract frequency-domain descriptors from the power spectrum, including high-frequency ratio, spectral centroid, cumulative-energy frequencies (*f*_50_,*f*_80_,*f*_95_), 1/*f* spectral slope, anisotropy ratio, angular entropy, noise floor, and an SNR-like measure. We then aggregated each descriptor across projections using robust statistics, such as median, percentiles, interquartile range and coefficient of variation, yielding a fixed-length metric vector *m*. The whole risk scoring pipeline is illustrated in Figure S2.

##### Global risk (*risk*_*global*_)

We quantified outlierness using a robust z-normalisation computed over the entire dataset:

$$\tilde{z}\left(m_{k}\right)=\frac{m_{k}-\text{median}\left(m_{k}\right)}{\hat{\sigma_{\text{rob}}}\left(m_{k}\right)+\epsilon }\text{,}$$

Where *m*_*k*_ denotes the k-th aggregated frequency-domain statistic and $\hat{\sigma_{\text{rob}}}\left(\cdot \right)$ denotes a robust scale estimator based on the median absolute deviation.

We define *risk*_*global*_ as a signed linear combination of robustly normalised metrics:

$$\text{ris}k_{\text{global}}=\sum_{k\in P}\tilde{z}\left(m_{k}\right)-\sum_{k\in N}\tilde{z}\left(m_{k}\right)-\frac{1}{2}\sum_{k\in Q}\tilde{z}\left(m_{k}\right)\text{,}$$

where *P* denotes a set of metrics whose larger robust deviations indicate higher structural irregularity (risk-increasing terms), *N* denotes metrics that capture within-sample consistency and are negatively correlated with risk (risk-decreasing terms), and *Q*denotes instability indicators for which we apply an additional penalty to emphasise projection-level variability. Larger *risk*_*global*_ indicates stronger deviation from the typical frequency profile and reduced projection-level consistency.

##### Predicted risk (*risk*_*pred*_)

While *risk*_*global*_ is annotation-free, we additionally learned a calibrated predictor of human-algorithm discrepancy. Given manual counts *n*_*human*_ and algorithmic instance counts *n*_*watershed*_, we defined the supervision signal

$$y=\left|\;n_{watershed}-n_{human}\;\right|$$

and train a lightweight Multi-Layer Perceptron regressor *f*_*θ*_ on the same standardised metric vector *m*.

$$\text{ris}k_{\text{pred}}=f_{\theta }\left(m\right)\approx \left|n_{watershed}-n_{human}\right|\text{.}$$

We implemented *f*_*θ*_ as an adaptive-weight model that outputs a sample-specific feature weighting and a scalar risk estimate and validate it using cross-validation. High *risk*_*pred*_ flags samples that are likely to require manual verification or parameter adjustment.

#### Automated antennal lobe, glomerular and whole-brain volume estimation

Following watershed segmentation, antennal lobe volumes were estimated using a 3D convex hull–based approach. For each segmented antennal lobe, all foreground voxel coordinates were extracted from the instance-labelled volume to form a point cloud, from which a convex hull was computed. To obtain a closed representation of the structure, small surface discontinuities were repaired to generate a watertight mesh. The resulting convex hull was then rasterised into a binary volume with the same spatial dimensions as the original image stack, such that all voxels enclosed by the hull were assigned as foreground. Antennal lobe volume was calculated by summing the number of foreground voxels and multiplying by the voxel volume, derived from the image metadata (xy pixel size and z-step size, in micrometres). This approach captures the overall morphological envelope of each antennal lobe and mitigates underestimation arising from internal gaps or incomplete segmentation of individual substructures. In addition to convex hull–based volume estimation, the volume of each glomerulus was quantified directly from the watershed instance segmentation. For each instance-labelled glomerulus, the total number of voxels assigned to that label was summed across the 3D volume. Glomerular volume was then computed by multiplying the voxel count by the voxel volume, derived from the xy pixel spacing and z-step size obtained from the microscopy metadata. This instance-based measurement provides a direct estimate of individual glomerular volumes and enables quantitative comparisons at the sub-structure level within each antennal lobe.

Whole brain volumes were estimated using the same intensity normalisation, contrast enhancement and initial binary segmentation procedures described for the 3D watershed-based antennal lobe segmentation. Briefly, confocal image stacks were standardised across samples, contrast-enhanced, and thresholded to obtain a coherent three-dimensional brain mask. Unlike the instance-level watershed segmentation applied to antennal lobe glomeruli, whole brain volume estimation did not require marker extraction or watershed partitioning. Instead, the binary brain mask was refined using 3D morphological operations to correct small gaps, irregular boundaries and internal cavities, followed by connected component analysis to remove residual noise. The largest connected component was retained as the whole brain mask. Whole brain volume was calculated by summing all voxels within the final binary mask and multiplying the voxel volume, derived from the xy pixel spacing and z-step size provided by the microscopy metadata.

#### Scanning Electron Microscopy

Scanning Electron Microscopy was conducted through a scanning electron microscope (SEM) Zeiss LEO 1430 (Oberkochen, Germany) and a SEM JEOL Ltd. (Tokyo Japan), both located at the University of Milan. The study was conducted on a total of 45 individuals (3–5 per sex and species). The heads of the ethanol stored bumblebees were delicately detached from the rest of the body and the antennae detached from the head. One antenna per each individual was then mounted, in a dorsal-lateral position, on aluminum stubs through an adhesive carbon tape and were subsequently platinum-plated using a sputter (EM ACE600, LEICA). We took SEM images of the pre-distal flagellomere at 120–180× magnification. The focus on the pre-distal flagellomere is justified by the fact that in bees the number of sensilla increases along the antenna.^58^^,^^59^ We preferred to focus on the pre-distal flagellomere since the distal one is sometimes curve or narrow near the tip of the antenna, but the pre-distal flagellomere has a more uniform shape across different individuals within species. A series of images were taken at greater magnification (300–500 X) of a large part of the pre-distal flagellomere. The SEM analysis was done in high vacuum (53.3–66.6 Pa) conditions, with resolution: 3.0 nm at 30 kV (secondary electrons), 10 nm at 3 kV (secondary electrons), and 4.0 nm at 30 kV (back-scattered electrons). The acceleration voltage was of 20–26 kV and the working distance was 10 mm. Stub-mounted antennae are deposited at the University of Milan.

#### Sensilla characterisation

The following measurements and counts were taken from the acquired SEM images, using the software ImageJ.^60^ We measured the length (FL) and the width (FW) of the pre-distal flagellomere (F9 for females, F10 for males) and obtained the flagellomere area (FL × FW). For the sensillar types, we primarily followed the classification proposed for Hymenoptera, including bumblebees,^61^^,^^62^^,^^63^^,^^64^ as well as the Hymenoptera Anatomy Ontology (HAO) portal^53^ (http://glossary.hymao.org/projects/32/public/ontology/). We quantified three main types of sensilla: the placoid sensilla (SP), the basiconic sensilla (SB) and the trichoid sensilla (ST) in a 100 × 100 μm square drawn in a region of the flagellomere visibly rich in SP,^41^^,^^59^ and we measured the area of five SP in each antenna, calculating subsequently a mean value per individual across these value. The three target sensillar types are easily distinguishable. The SP have a flat appearance, they are elongated and rich in pores. The SB are hair-like, short and thick pored sensilla with a grooved surface, with their pegs arising from a shallow socket, and they are generally not curved. Trichoid sensilla have also the shape of a hair but they are thinner and longer than SB, are often curved and may have an apical pore; they are generally divided into sub-types,^65^^,^^66^^,^^67^ that were here pooled together since at our working accuracy with SEM it was not possible to go deeper in their ultrastructure. Based on previous histological and/or physiological studies,^68^^,^^69^ SP have an olfactory function, SB an olfactory or gustatory function, and ST are either mechanoreceptors or taste receptors, depending on whether they are not pored or pored, respectively. Ultrastructural studies showed that they function as mechanoreceptors in Apidae,^58^ though some sub-types may be porous and acting as chemoreceptors.^58^ Hence, we here considered SP and SB, rather than ST, as the most relevant olfactory sensilla and their densities were summed in subsequent statistical analyses. However, ST were also tested for sex- and species-specific differences to obtain a complete description.

### Quantification and statistical analysis

#### Statistical analysis of (macro)glomeruli

K-statistics were used to identify enlarged/macroglomeruli within a sample. The K-value is calculated as:

$$K=\frac{\left(V_{G}-V_{U}\right)}{\left(V_{U}-V_{L}\right)}$$

where V_G_ is the volume of the focal glomerulus, V_L_ is the 10th percentile of the glomerular volume distribution, and V_U_ is the 90th percentile of the glomerular volume distribution. The K-value indicates how many times the focal glomerulus exceeds the upper percentile of the interpercentile range. Glomeruli with a K-value greater than 3 were classified as macroglomeruli, as seen in previous studies.^17^^,^^18^^,^^19^^,^^20^^,^^21^^,^^30^ Glomeruli with a K-value between 1.5 and 3 were, unless stated otherwise, classified as enlarged glomeruli or putative macroglomeruli.^20^^,^^30^ While a glomerulus was assigned to a quantitative size class exclusively from its K-value, a subthreshold glomerulus may be labeled separately as a putative anatomical homologue when it is consistently identifiable across conspecific samples and occupies the corresponding position of a threshold-positive glomerulus in another species. The same rule was applied across all six species. K-values were calculated using Microsoft Excel. “n” in Figure legends indicates the number of individual bees analyzed. One antennal lobe or one antenna was analyzed per bee.

#### Statistical analysis of anatomical traits

Analysis of how sex and species influence total brain volume (Figure 1), total and relative AL volume (Figure 1), number of glomeruli (Figure 2) and sensillar numbers (Figure 4) was conducted with 2-way Analysis of Variance (ANOVA). Simple liner regression models were used to correlate total and relative AL volume with total brain volume (Figure 1), automatic and manual measurements of the total AL volume (Figure 1), number of glomeruli and total AL volume (Figure 2), automatic and manual evaluations of the glomerular numbers (Figure 2), the number of glomeruli and sensillar density/number of plant species (Figure 4), and the total AL volume and the number of plant species (Figure 4). All statistical analyses were carried out using GraphPad Prism version 10.2.0.
