# Supplementary material for: Sexual dimorphisms of the olfactory system are conserved across bumblebee species
Source: iScience. 2026 Oct 28;29(11):118037. doi: 10.1016/j.isci.2026.118037 (PMC13738290; doi:10.1016/j.isci.2026.118037)
Supplement: Supplementary file 1 [file mmc1.pdf]

iScience, Volume *29*

Supplemental information

Sexual dimorphisms of the olfactory system are conserved across bumblebee species

Jorn Oppersma, Wenke E, W. Carolina Gomez-Ramirez, Iman Muktar, Timothy Wise, Miranda Bolton, Shuang Chen, Amir Atapour-Abarghouei, Carlo Polidori, and Olena Riabinina

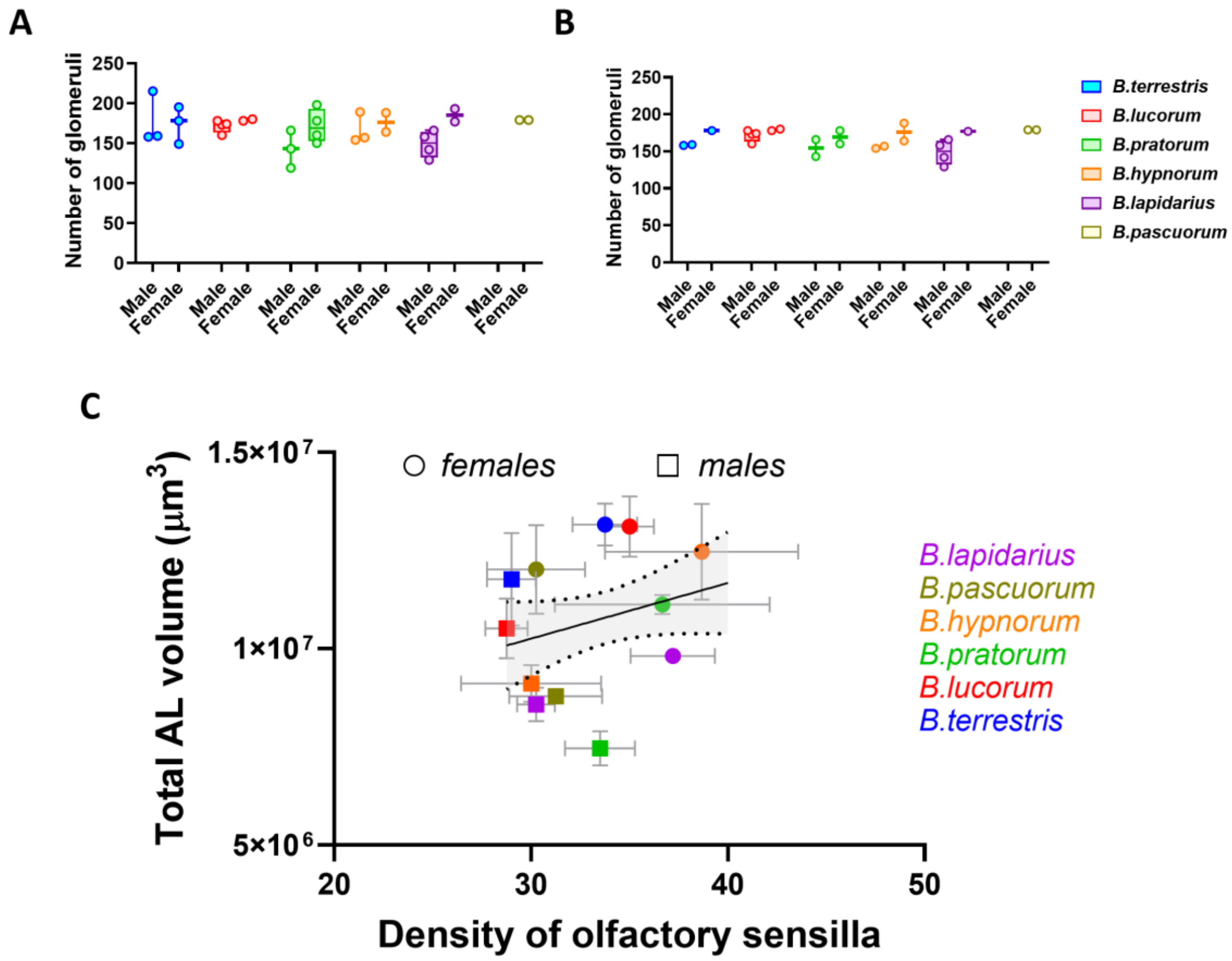

Figure S1: **Automated glomerular counts and the relationship between antennal lobe volume and olfactory sensillar density. A)** Automatically segmented (without risk-score, n=32) number of glomeruli in the AL of male (M) and female (F) of *B.terrestris* (M:n=3, F:n=3), *B.lucorum* (M:n=4, F:n=2), *B.pratorum* (M:n=3, F:n=4), *B.hypnorum* (M:n=3, F:n=2), *B.lapidarius* (M:n=4, F:n=2) and *B.pascuorum* (M:n=0, F:n=2). **B)** Automatically segmented (with risk-score, n=24) number of glomeruli in the AL of male (M) and female (F) of *B.terrestris* (M:n=2, F:n=1), *B.lucorum* (M:n=4, F:n=2), *B.pratorum* (M:n=2, F:n=2), *B.hypnorum* (M:n=2, F:n=2), *B.lapidarius* (M:n=4, F:n=1) and *B.pascuorum* (M:n=0, F:n=2). Box-whisker plots (**A, B**): the box indicates 25th, 50th and 75th percentiles, the whiskers extend to min and max values. **C)** Correlation of the total AL volume and the density of olfactory sensilla. The line and the shaded areas represent simple linear regression lines with 95% confidence interval. Error bars represent standard deviations.

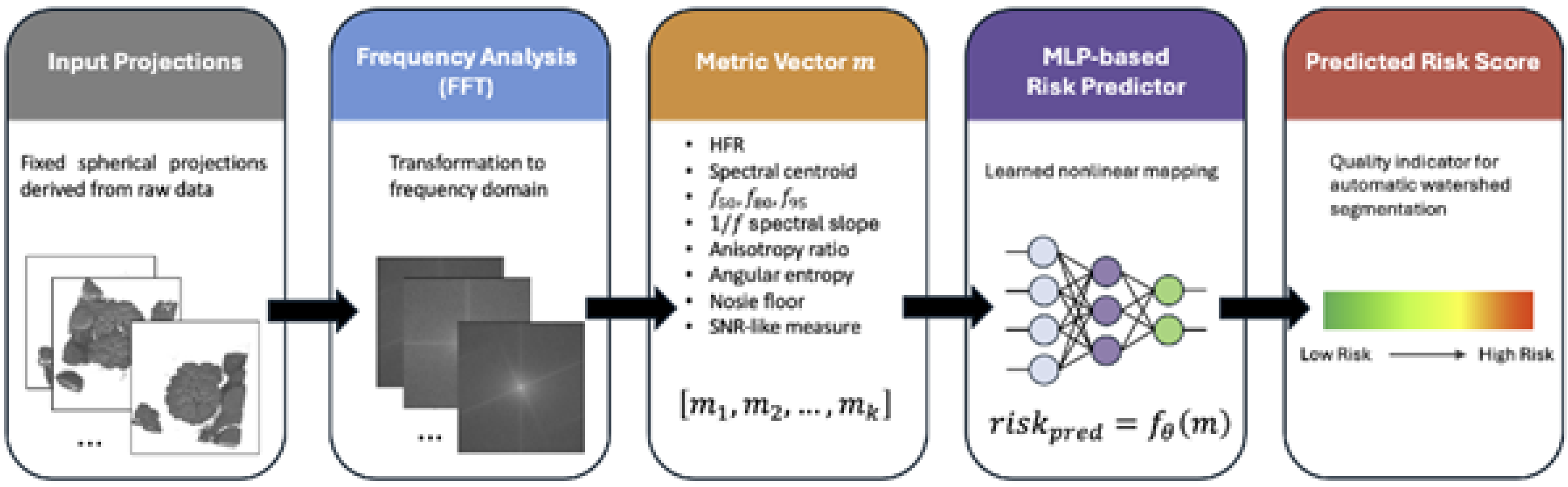

Figure S2: Schematic overview outlining the risk scoring pipeline.

Table S1: Plant species that this study's bumblebees were collected from.

Bumblebee species	Sex	Plant species	Total
Bombus hypnorum	Female	*Centaurea nigra, Cistus sp., Cotoneaster × suecicus, Epilobium angustifolium, Lapsana communis, Primula bulleyana, Tilia spp.*	7
Bombus hypnorum	Male	*Arctium minus, Cistus salvifolius, Heracleum sphondylium, Rubus fruticosus agg. Symphoricarpos albus, Tilia spp., Veronica albicans.*	7
Bombus lapidarius	Female	*Cardamine pratensis, Centaurea nigra, Cirsium arvense, Cirsium vulgare, Cistus salvifolius, Epilobium angustifolium, Impatiens glandulifera, Jacobaea vulgaris, Lapsana communis, Lavandula angustifolia, Linum usitatissimum, Lotus corniculatus, Ranunculus acris, Raphanus sativus, Rubus fruticosus agg., Tilia spp.*	16
Bombus lapidarius	Male	*Centaurea nigra, Cirsium arvense, Cirsium vulgare, Cistus criticus, Cyanus segetum, Epilobium angustifolium, Impatiens glandulifera, Jacobaea vulgaris, Lavandula angustifolia, Raphanus sativus, Salvia yangii, Veronica albicans.*	12
Bombus lucorum	Female	*Centaurea nigra, Cornus sanguinea, Epilobium angustifolium, Filipendula ulmaria, Heracleum sphondylium, Hydrangea paniculata, Hypericum perforatum, Jacobaea vulgaris, Rhinanthus minor, Rubus fruticosus agg., Tilia spp., Trifolium repens.*	12
Bombus lucorum	Male	*Centaurea nigra, Cirsium arvense, Cornus sanguinea, Epilobium angustifolium, Heracleum sphondylium, Lavandula angustifolia, Ligustrum vulgare, Phacelia tanacetifolia, Salvia yangii, Tilia spp.*	10
Bombus pascuorum	Female	*Ballota nigra, Calystegia silvatica, Centaurea nigra, Cirsium arvense, Cornus sanguinea, Cyanus segetum, Echium pininana, Epilobium angustifolium, Geranium pratense, Hypericum perforatum, Impatiens glandulifera, Lathyrus pratensis, Lavandula angustifolia, Ligustrum vulgare, Linum usitatissimum, Lotus corniculatus, Nepeta racemosa, Odontites sp., Phacelia*	32

		tanacetifolia, Primula bulleyana, Raphanus sativus, Rhinanthus minor, Rhododendron spp., Rubus caesius, Rubus fruticosus agg., Salvia rosmarinus, Salvia yangii, Sinapis alba, Stachys palustris, Trifolium pratense, Trifolium repens, Vicia cracca	
Bombus pascuorum	Male	*Ballota nigra, Calystegia silvatica, Centaurea nigra, Cirsium arvense, Impatiens glandulifera, Lavandula angustifolia, Rhinanthus minor, Rubus fruticosus agg., Vicia cracca.*	9
Bombus pratorum	Female	*Campanula latifolia, Centaurea nigra, Epilobium angustifolium, Geranium pratense, Impatiens glandulifera, Lavandula angustifolia, Rhinanthus minor, Rhododendron spp., Salvia yangii, Symphoricarpos albus, Tilia spp.*	11
Bombus pratorum	Male	*Arctium minus, Cirsium arvense, Cistus salvifolius, Geranium pratense, Ligustrum vulgare, Ranunculus acris, Rhododendron sp1., Rhododendron sp2., Rubus fruticosus agg., Salvia rosmarinus, Symphoricarpos albus, Tilia spp.*	12
Bombus terrestris	Female	*Buddleja davidii, Centaurea nigra, Cirsium arvense, Cirsium vulgare, Cistus salvifolius, Cotoneaster × suecicus, Cyanus segetum, Epilobium angustifolium, Filipendula ulmaria, Heracleum sphondylium, Hydrangea paniculata, Hypericum perforatum, Impatiens glandulifera, Jacobaea vulgaris, Lavandula angustifolia, Ligustrum vulgare, Nepeta racemosa, Papaver rhoeas, Raphanus sativus, Silene dioica, Symphoricarpos albus, Trifolium repens.*	22
Bombus terrestris	Male	*Buddleja davidii, Calystegia silvatica, Centaurea nigra, Cirsium arvense, Cistus sp., Epilobium angustifolium, Impatiens glandulifera, Jacobaea vulgaris, Lavandula angustifolia, Ligustrum vulgare, Nepeta racemosa, Phacelia tanacetifolia, Rhododendron ponticum, Rubus caesius, Rubus fruticosus agg., Salvia yangii, Tilia sp1., Tilia sp2., Veronica spicata.*	19
